# Characterization of the intestinal fungal microbiome in patients with hepatocellular carcinoma

**DOI:** 10.1186/s12967-023-03940-y

**Published:** 2023-02-15

**Authors:** Lilong Zhang, Chen Chen, Dongqi Chai, Chunlei Li, Zhendong Qiu, Tianrui Kuang, Li Liu, Wenhong Deng, Weixing Wang

**Affiliations:** 1 Department of General Surgery, Renmin Hospital of Wuhan University, No.238, Jiefang Road, Wuchang District, Wuhan, 430060 Hubei China; 2 Hubei Key Laboratory of Digestive System Disease, No.238, Jiefang Road, Wuchang District, Wuhan, 430060 Hubei China; 3 Central Laboratory, Renmin Hospital of Wuhan University, No. 238, Jiefang Road, Wuchang District, Wuhan, 430060 Hubei China

**Keywords:** Gut mycobiome, Hepatocellular carcinoma, Liver cirrhosis, ITS2 rDNA sequencing, *Malassezia*, *Candida albicans*

## Abstract

**Objective:**

Gut mycobiota plays a crucial role in benign liver diseases; however, its correlation with hepatocellular carcinoma (HCC) remains elusive. This study aimed to elucidate fungal differences in patients with HCC-associated cirrhosis compared to cirrhotic patients without HCC and healthy controls.

**Methods:**

The 72 fecal samples from 34 HCC patients, 20 cirrhotic patients, and 18 healthy controls were collected and analyzed using ITS2 rDNA sequencing.

**Results:**

Our results revealed the presence of intestinal fungal dysbiosis with significant enrichment of opportunistic pathogenic fungi such as *Malassezia*, *Malassezia *sp., *Candida*, and *C. albicans* in HCC patients compared with healthy controls and cirrhosis patients. Alpha-diversity analysis demonstrated that patients with HCC and cirrhosis showed decreased fungal diversity compared to healthy controls. Beta diversity analysis indicated that the three groups exhibited significant segregated clustering. Besides, *C. albicans* was found to be significantly more abundant in the HCC patients with TNM stage III-IV than those with stage I-II, in contrast to the commensal organism *S. cerevisiae*. We also confirmed that the HCC patients were successfully classified with an area under the curve value of 0.906 based on the fecal fungal signature. Finally, our animal experiments confirm that aberrant colonization of the intestine by *C. albicans* and *M. furfur* can promote the development of HCC.

**Conclusions:**

This study indicates that dysbiosis of the gut mycobiome might be involved in HCC development.

*Trial registration*: ChiCTR, ChiCTR2100054537. Registered 19 December 2021, http://www.chictr.org.cn/edit.aspx?pid=144550&htm=4

**Supplementary Information:**

The online version contains supplementary material available at 10.1186/s12967-023-03940-y.

## Introduction

Primary liver cancer is the sixth most prevalent malignancy and the third leading cause of cancer-related mortality worldwide [1]. Hepatocellular carcinoma (HCC) is the most common type of liver cancer, accounting for 80% of cases [2]. Despite recent advances in HCC therapy, the 5 year survival rate of HCC patients remains dismal due to the spread, metastases, and high rate of recurrence [3, 4]. Besides, the initial symptoms of HCC are insidious, and more than half of the HCC patients are already at an advanced stage when diagnosed, thereby missing the best treatment options [4–6]. Consequently, there is an urgent need to explore the mechanisms of HCC development and identify novel diagnostic markers to improve prognosis.

Currently, an increasing number of studies have noted the association between gut microbiome dysbiosis and HCC development [7–11]. The term “microbiome” refers to microorganisms, including bacteria, fungi, protozoa, parasites, and viruses. However, the vast majority of studies have focused on intestinal bacteriome, and few studies have investigated the correlation between gut fungi and HCC. This may be because the intestinal bacteriome accounts for over 99% of the gut microbiome [12]. In reality, fungi have unique and intriguing properties that set them apart from other microbiomes. An average fungal cell is about 100-fold larger than an average bacterial cell providing abundant bioactive molecules to the host and shaping its physiology [13, 14]. Furthermore, fungi are morphologically versatile, and able to transition from unicellular (like yeast) to multicellular (such as during hyphal growth), affecting their pathogenicity. Normally, hyphae are more invasive, while yeasts are non-invasive and frequently commensal [15]. Fungi also produce a wide range of harmful and beneficial compounds. Toxic metabolites derived from fungi, also known as mycotoxins, remain in the organism and cause damage even after the fungi have been eradicated [16]. Aflatoxins (mycotoxins produced by secondary metabolism of the fungus *Aspergillus flavus* and *Aspergillus parasiticus*) are known to induce HCC [17]. In addition to the carcinogenic role of mycotoxins, several studies have shown that the intestinal mycobiota is directly involved in the development of colorectal, oral, and pancreatic cancer [18–26], and multicentre clinical research has revealed that gut fungi can be used for the diagnosis of colorectal cancer [19].

The liver and gut have a bidirectional connection through the portal vein and biliary system [27]. Potential antigens derived from gut fungi can penetrate the gastrointestinal barrier and translocate to the liver via the portal vein, compromising its function. To maintain homeostasis, immune cells that reside in or travel through the liver initiate innate and/or adaptive immune responses against gut mycobiome [28]. Recently, there have been many exciting discoveries about the roles of gut mycobiota in benign liver disease [28–32]. Nevertheless, little is known about the gut fungal composition in HCC patients. Thus, it is necessary to perform further studies regarding the changes in the composition of gut fungi in the progression from health, cirrhosis to HCC, thereby providing a new theoretical basis for the prevention and treatment of HCC.

## Materials and methods

### Participant recruitment

The HCC-cirrhosis patients, liver cirrhosis (without HCC), and healthy controls were prospectively recruited at the Renmin Hospital of Wuhan University from Dec 2021 to Jul 2022. This research was performed in conformity with the Helsinki Declaration and Rules of Good Clinical Practice. It was approved by the Clinical and Animal Research Ethics Committee of Renmin Hospital of Wuhan University and registered on the Chinese Clinical Trial Registry Platform (Registry ID: ChiCTR2100054537). All participants signed a written informed consent form. The demographic data and clinical characteristics of patients were gathered through medical records and direct interviews.

The HCC patients were diagnosed by histopathological examination of specimens from surgical resection or percutaneous ultrasound-guided liver needle core biopsy. Mixed-type liver cancer (hepatocellular carcinoma and intrahepatic cholangiocarcinoma) was excluded. The diagnosis of cirrhosis was based on the comprehensive integration of histological examination, imaging findings, laboratory parameters, clinical symptoms, physical signs, and medical history. Before stool sample collection, HCC patients had not received any standard anti-tumor therapy. The healthy controls were selected from the people who came to our hospital for a medical examination.

Individuals with other malignancies, digestive system diseases, autoimmune diseases, and acute or chronic infectious diseases (except for hepatitis B viral (HBV) infection) were excluded. Besides, all included subjects reported no probiotics or prebiotics use, no antibiotic or antifungal use, and no laxatives within 3 months.

### Sample collection, DNA extraction, PCR amplification, and ITS2 sequencing

A fecal sample was collected by the participants, transported to the laboratory within 2 h, and stored at − 80 °C until total DNA was extracted. The CTAB method was applied to extract total DNA from stool samples [33]. Purity and concentration of DNA were measured by agarose gel electrophoresis (1% agarose gels). The hypervariable ITS2 region of the ITS gene was amplified using a specific primer with the barcode. Detailed steps for PCR amplification can be found in the Additional file 1. Mix the same volume of 1 × loading buffer (contained SYB green) with the PCR products and operate electrophoresis on 2% agarose gels for detection. The Qiagen Gel Extraction Kit (Qiagen, Germany) was then used to purify the PCR products in the mixture. Following the manufacturer’s instructions, sequencing libraries were created using the TruSeq^®^ DNA PCR-Free Sample Preparation Kit (Illumina, USA), and index codes were added. The Qubit@ 2.0 Fluorometer (Thermo Scientific) was utilized to assess the library’s quality. Finally, on an Illumina Novaseq 6000 PE250 platform, the library was sequenced and 250 bp paired-end reads were generated.

### Bioinformatics analysis

The NCBI Sequence Read Archive database was used to store the raw sequencing data for all samples (accession number, PRJNA887395). The raw sequencing data were reads spliced, tags filtered and chimera removed to obtain effective tags (Additional file 2: Table S1), then OTUs cluster and species annotation were conducted (Additional file 3: Table S2). Detailed steps were described in the Additional file 1. A Venn diagram was created via the R (Version 2.15.3) package “VennDiagram” to visualize the shared and unique OTUs among the three groups. The rank abundance curves were displayed using R software. The alpha diversity (Shannon and Simpson) and beta diversity on weighted_unifrac distances were calculated using QIIME (Version 1.9.1). The principal co-ordinates analysis (PCoA) analysis was completed using the WGCNA, stats, and ggplot2 packages of R software [34], and non-metric multidimensional scaling (NMDS) analysis was performed using the vegan package of R software [35]. For multiple response permutation procedure (MRPP) analysis, the MRPP function of the vegan package for R was applied [36]. The linear discriminant analysis effect size (LEfSe) analysis was carried out using the LEfSe software (Version 1.0) with a default setting of 4 for the linear discriminant analysis (LDA) score screening [37]. The Spearman correlation coefficient values for taxa and clinical physiological indicators were calculated for significance using the corr.test function in the psych package, and then visualized using the pheatmap package of R software. The clinical parameters with a strong influence on the flora were screened by variance inflation factor (VIF) analysis and BioENV analysis in turn, and then subjected to canonical correspondence analysis (CCA) [38, 39]. To further explore the impact of mycobiota community change, functional prediction with FUNGuild annotation tools was deployed [40]. The receiver operating characteristic (ROC) curve was used to explore the potential ability of intestinal fungi to discriminate between 34 HCC patients and 38 non-HCC subjects (cirrhotic patients and healthy controls). The prediction performance of the fungal microbes was evaluated by the area under the curve (AUC). The combined model was estimated to use a binary logistic regression model (enter method) to calculate predicted HCC probabilities and plot ROC curves using IBM SPSS Statistics 26.

### Cell culture and strain culture

Hepa1-6 cells (hepatocellular carcinoma cell line) were obtained from Wuhan University. The cells were cultured in Dulbecco’s modified Eagle medium (Servicebio, Wuhan, China) with 1% penicillin-streptomycin (Biosharp, Hefei, China) and 10% fetal bovine serum (Gibco, Grand Island, NY, United States) at 37 °C with 5% CO2. The *Candida albicans* SC5314 standard strain was purchased from Biofeng Lab (Shanghai, China) and inoculated in yeast extract-peptone-D-glucose (YPD, Hopebio, Qingdao, China) and cultured for 24 h with continuous shaking at 30 °C overnight. The *Malassezia furfur* ATCC14521 strain was purchased from Fenghui Biotechnology Co., Ltd (Changsha, China) and inoculated in 2693 Modfied Dxn (mDxan, Hopebio, Qingdao, China) and cultured for 48 h with continuous shaking at 30 °C overnight.

### Animal experiments

Male C57BL/6 mice were purchased from SHULAIBAO BIOTECHNOLOGY (Wuhan, China). Mice were given Cefoperazone (1 mg/mL) in the water for up to one week following a week of acclimation, which can promote robust *C. albicans* and *M. furfur* colonization [41] with limited impact on the bacterial microbiome in the absence of colonization [42]. Then, the mice were randomized to *C. albicans*, *M. furfur*, and control groups (6 mice per group). In both experimental groups, *C. albicans* and *M. furfur* were administered by oral gavage at a dose of 4 × 10^8^ CFUs in sterile phosphate-buffered saline (PBS, 0.2 ml). The control mice received gavage with PBS (0.2 ml) only. The gavage was performed every other day for 5 weeks. After the fungal gavage lasting 3 weeks, 1 × 10^7^ Hepa1-6 cells were inoculated subcutaneously into the left flank of mice, and the mice were euthanized 2 weeks later. Tumor volume was assessed according to the formula: volume = (tumor length × tumor width^2^) × 0.52 [43].

### Immunohistochemical staining

Mouse tumor tissue was collected for paraffin embedding. Paraffin sections were baked, dewaxed, hydrated, antigenically repaired, blocked, and incubated overnight with the anti-Ki67 antibody (Rabbit, abclonal, 1:100), then incubated with a second antibody. Images were obtained using the fluorescence microscope (Olympus BX63, Tokyo, Japan). See the Additional file 1 for detailed immunohistochemical staining procedures.

### Statistical analysis

The categorical variables were compared using Fisher's exact test. The Wilcoxon rank-sum test was used to compare continuous variables between the two groups. A two-sided *P*-value < 0.05 indicated a significant difference.

## Results

### Characteristics of the participants

Following a rigorous pathological diagnosis and exclusion process, 72 fecal samples from 34 HCC-cirrhosis patients, 20 cirrhosis patients, and 18 healthy controls were collected and analyzed using ITS2 rDNA sequencing. Benefiting from a uniform sample collection protocol, all stool samples were yellow and soft. The clinical characteristics of the participants, such as age, gender, and body mass index (BMI), were matched among the three groups (Table 1). The patients with HCC and cirrhosis also have similar Child-Pugh and cirrhotic etiological compositions. Serum levels of alanine aminotransferase (ALT), aspartate aminotransferase (AST), total bilirubin (TBIL), and direct bilirubin (DBIL) were significantly elevated, whereas albumin and platelet levels were significantly reduced in HCC patients compared with healthy controls (Table 1). Compared to the cirrhosis group, the albumin level was significantly lower in the HCC group, while the remaining serum parameters were not significantly different (Table 1).

**Table 1:** Clinical characteristics of the enrolled participants

Parameter	Healthy controls (n = 18)	Cirrhosis (n = 20)	HCC-cirrhosis (n = 34)	*P-*values^a^	*P-*values^b^
Age (year)	57.5 (46.6–64.0)	57.0 (53.0–61.0)	57.5 (48.5–66.5)	0.672	0.830
BMI (kg/m^2^)	22.8 (20.7–25.4)	23.0 (21.0–24.9)	23.1 (20.5–24.0)	0.610	0.929
Gender					
Female	3 (16.67%)	4 (20.00%)	7 (20.59%)	1.00	1.00
Male	15 (83.33%)	16 (80.00%)	27 (79.41%)		
Cirrhosis etiology					
Hepatitis B virus	–	16(80.00%)	27 (79.41%)	–	1.00
NAFLD	–	4 (20.00%)	7 (20.59%)		
Child–Pugh					
A	–	18 (90.00%)	31 (91.18%)	–	1.00
B	–	2 (10.00%)	3 (8.82%)		
ALT (9-50U/L)	24.0 (18.5–31.1)	33.0 (26.3–40.8)	34.0 (19.8–56.3)	**0.043**	0.687
AST (15-40U/L)	26.3 (22.0–31.3)	38.5 (30.0–51.0)	43.5 (33.5–107.8)	** < 0.001**	0.117
Albumin (40-55 g/L)	43.6 (42.0–47.6)	42.3 (38.0–47.2)	37.4 (33.4–42.3)	** < 0.001**	**0.003**
Globulin (20-40 g/L)	26.0 (22.7–30.7)	27.7 (26.2–29.3)	26.9 (24.8–32.1)	0.281	0.740
TBIL (0-23 μmol/L)	13.7 (12.8–19.0)	16.3 (13.2–19.7)	20.9 (14.3–25.2)	**0.035**	0.144
DBIL (0-8 μmol/L)	6.20 (4.2–7.2)	6.8 (5.5–11.9)	8.3 (5.1–11.4)	**0.022**	0.957
PT (9-13 s)	–	11.4 (10.5–12.5)	12.1 (11.0–13.0)	–	0.147
Platelet (125–350 10E9/L)	229.5 (204.6–252.2)	193.0 (173.0–241.0)	175.0 (122.0–236.8)	**0.015**	0.457
Dietary habit	Mixed diet	Mixed diet	Mixed diet	**–**	**–**

Bold represents *P* value less than 0.05

Median (interquartile range) or *n* (%).Continuous variables were compared using Wilcoxon rank-sum test between both groups. Categorical variables were compared using Fisher’s exact test

*HCC* hepatocellular carcinoma, *BMI* body mass index, *ALT* alanine aminotransferase, *AST* aspartate aminotransferase, *TBIL* total bilirubin, *DBIL* direct bilirubin, *PT* prothrombin time, *NAFLD*, non-alcoholic fatty liver disease

^a^HCC patients vs healthy controls

^b^HCC patients vs cirrhosis-only patients

### Altered community composition in HCC patients compared to healthy controls and hepatocirrhosis patients

**Fig. 1:**
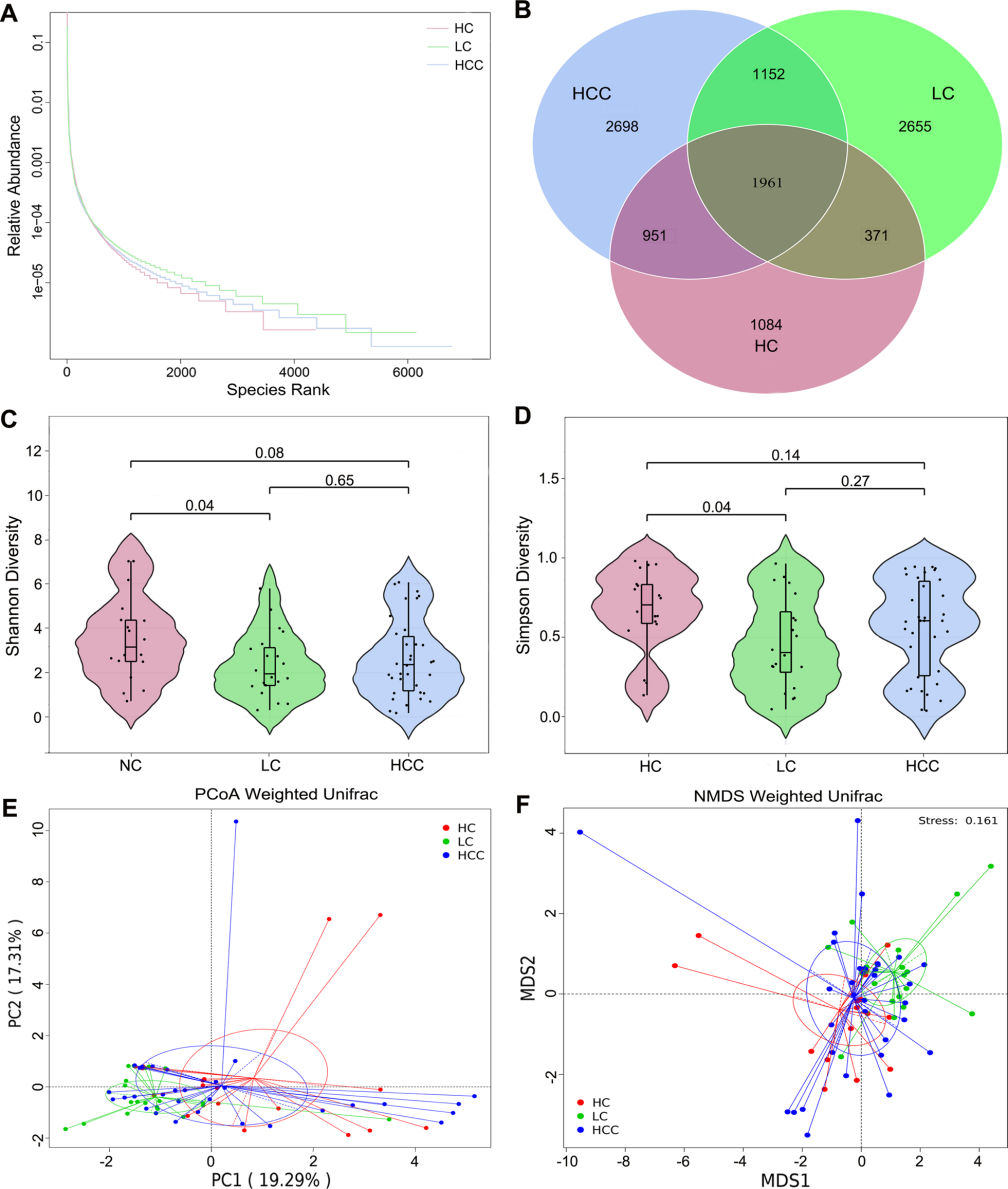
Diversity analysis in the HCC, LC, and HC groups. **A** Rank abundance curves. **B** Venn diagram displaying the overlap of OTUs identified among the three groups. Alpha diversity was estimated by the Shannon index **C** and Simpson index **D**. The distributional difference of gut mycobiota profiles was assessed using PCoA **E** and NMDS **F** based on a weighted_unifrac matrix. *HCC* hepatocellular carcinoma, *LC* liver cirrhosis, *HC* healthy controls

First, we assessed the composition of the gut fungi in our cohort. The rank abundance curves revealed that the species have good richness and uniformity in each group (Fig. 1A). The Venn diagram showed that 1961 OUTs were shared among the three groups. 2698 OTUs, 2655 OTUs, and 1084 OTUs were unique to the HCC group, cirrhosis group, and healthy controls, respectively, implying that HCC patients have the greatest abundance of unique OTUs (Fig. 1B). The fungal alpha diversity, as estimated by the Shannon diversity and Simpson diversity, was significantly reduced in patients with cirrhosis compared to healthy controls (*P* = 0.04 and 0.04 respectively; Fig. 1C, D). The above indicators also displayed a decreasing tendency in fungal diversity from the healthy controls to the HCC patients, but the difference was not significant (*P* = 0.08 and 0.14, respectively; Fig. 1C, D). However, there was no significant difference in diversity between the two patient cohorts (*P* = 0.65 and 0.25, respectively; Fig. 1C, D).

The beta diversity was next applied to explore the fungal compositions among the three groups by performing weighted_unifrac matrix. The PCoA showed that the three groups of individuals formed a fairly good separation of gut fungi (Fig. 1E). In addition, NMDS is applied to visualize the distances among the three groups, as shown in Fig. 1F, illustrating the distinct separation. MRPP analysis further confirmed the above findings (HCC vs. cirrhosis: *P* = 0.048; HCC vs. control: *P* = 0.041; cirrhosis vs. control: *P* = 0.021). Taken together, these results indicate the presence of altered intestinal fungal composition in patients with HCC.

### Gut fungal dysbiosis in HCC-cirrhosis, including increased levels of C. albicans and Malassezia sp.

**Fig. 2:**
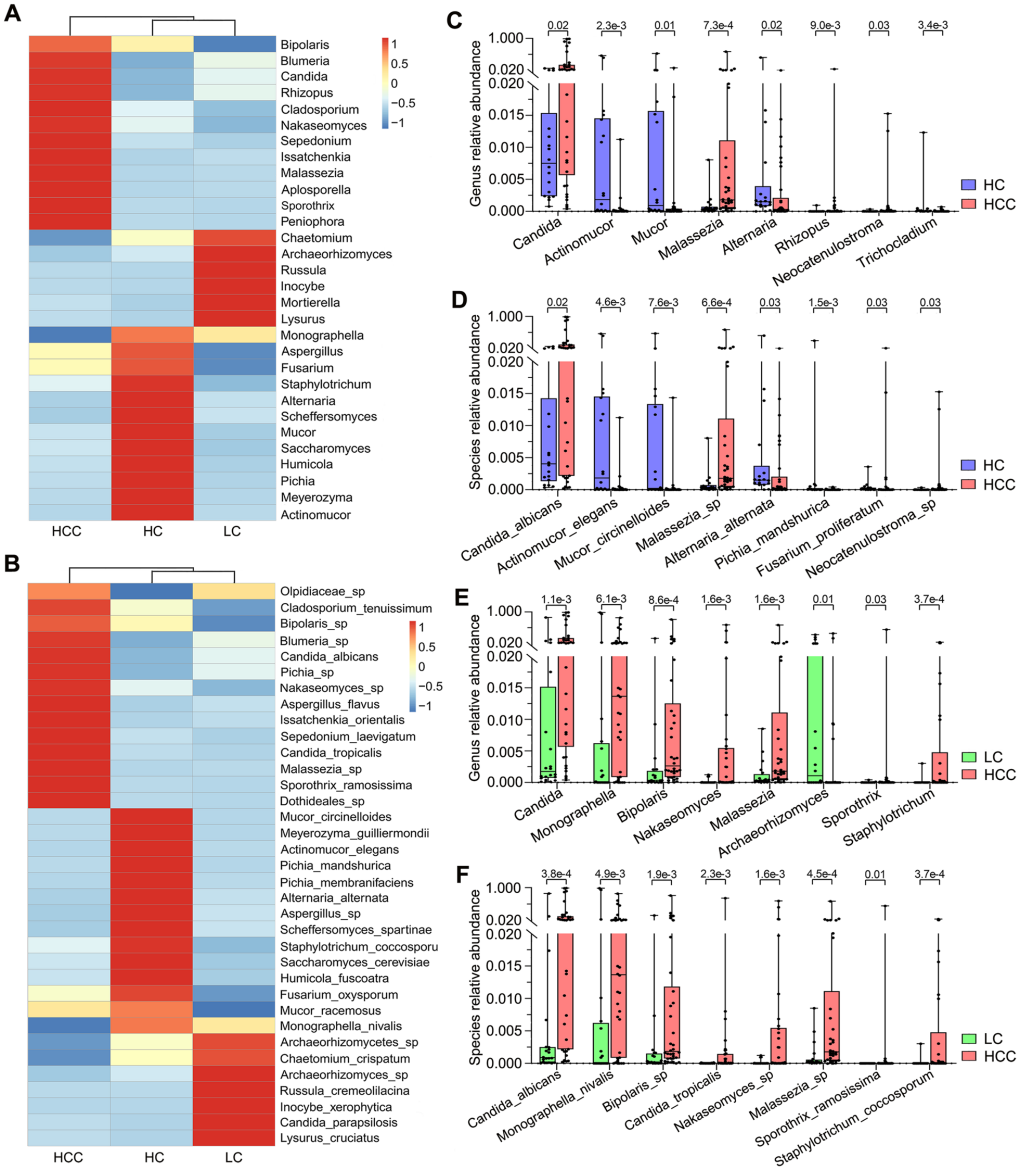
Differential analysis of fungal communities in the HCC, LC, and HC groups. **A** Heatmap of fungal abundance clustering at the genus level **A** and species level **B**. The eight most abundant of the differential taxonomic units at the genus level **C** and species level **D** between HCC patients and healthy controls. The eight most abundant of the differential taxonomic units at the genus level **E** and species level **F** between HCC patients and cirrhosis patients. The Wilcoxon rank-sum test was used. *HCC* hepatocellular carcinoma, *LC* liver cirrhosis, *HC* healthy controls

As shown in Fig. 2A, B, the cluster heatmap showed the differentially enriched fungal microbiotas among the three groups at the genus and species levels. Next, the Wilcoxon rank-sum test was used to explore differences at the genus and species levels. The results showed that there were 32 and 47 significantly differential taxa at the genus and species levels between HCC patients and healthy controls (Additional file 4: Table S3). The eight most abundant of the above differential taxonomic units were presented in Fig. 2C, D. We found that the relative abundances of *Candida*, *C. albicans*, *Malassezia*, *Malassezia *sp., *Rhizopus*, *Neocatenulostroma*, *Neocatenulostroma *sp., and *F. proliferatum* were significantly higher in HCC patients compared to healthy controls, while the relative abundances of *Actinomucor*, *A. elegans*, *Mucor*, *M. circinelloides*, *Alternaria*, *A. alternata*, *Trichocladium*, and* P. mandshurica* were significantly lower (Fig. 2C, D). We further analyzed the fungal differences between HCC patients and cirrhosis patients, finding a significant enrichment of *Candida*, *C. albicans*, *C. tropicalis*, *Monographella*, *M. nivalis*, *Bipolaris*, *Bipolaris *sp., *Nakaseomyces*, *Nakaseomyces *sp., *Malassezia*, *Malassezia *sp.,* Sporothrix*, *S. ramosissima*, *Staphylotrichum*, *S. coccosporum* and depletion of *Archaeorhizomyces* in the HCC group (Fig. 2E, F, Additional file 5: Table S4). These data point to the presence of fungal dysbiosis in HCC patients, which might be linked to hepatocellular carcinogenesis.

**Fig. 3:**
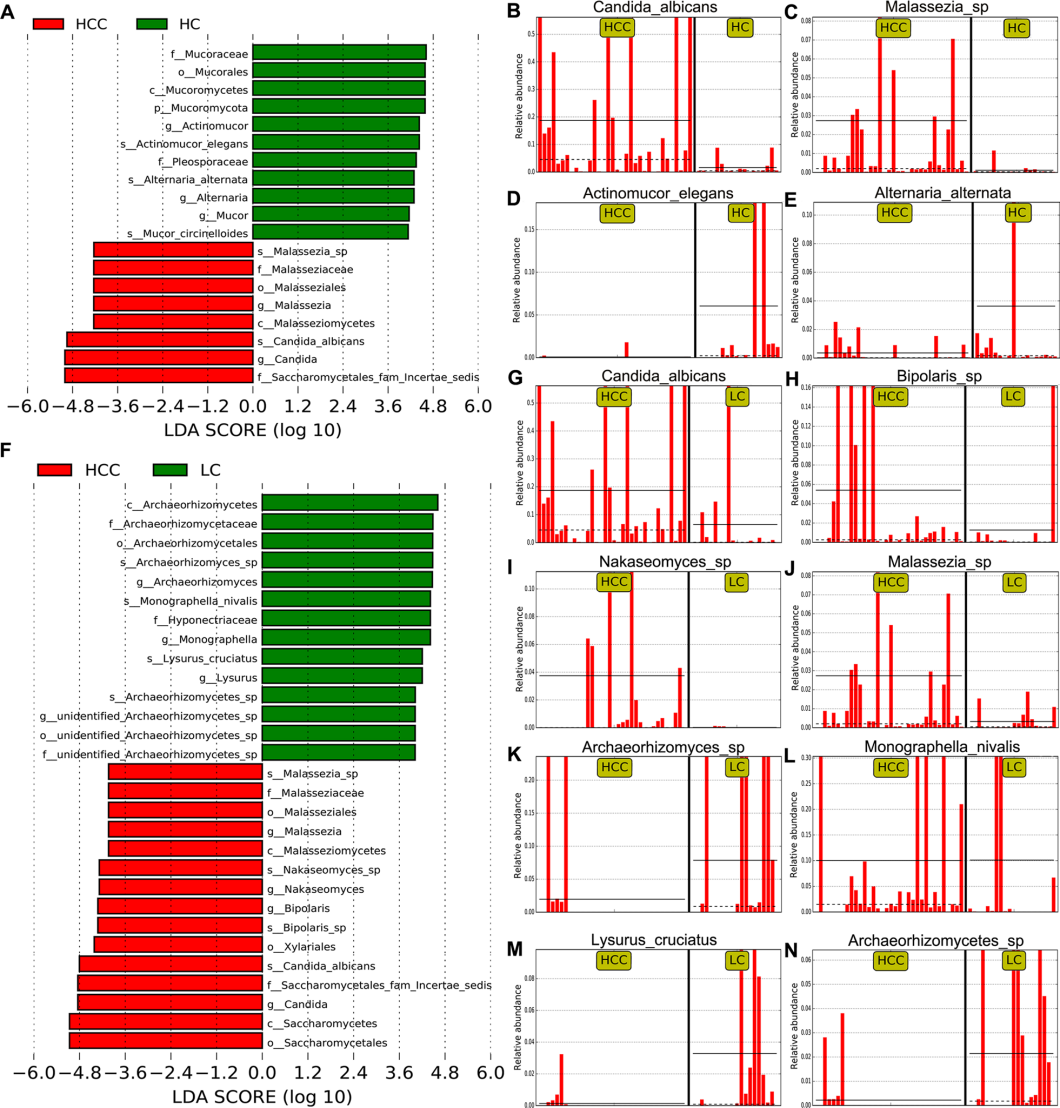
The differential taxa in the HCC, LC, and HC groups using the linear discriminant analysis effect size (LEfSe) analysis. **A** LDA scores were computed for differentially abundant taxa in the gut fungi of HCC patients and healthy controls. **F** LDA scores were computed for differentially abundant taxa in the gut fungi of HCC patients and cirrhosis patients. Length indicates the effect size associated with a taxon. *P* = 0.05 for the Kruskal-Wallis sum-rank test; LDA score > 4. **B**–**E**, **G**–**N** The histogram of the relative abundance distribution of each taxon at the species level. *HC* healthy controls, *HCC* hepatocellular carcinoma, *LC* liver cirrhosis, *LDA* linear discriminant analysis

To identify the specific fungal taxa associated with HCC, we further compared the fungal composition between the HCC patients and healthy controls using LEfSe. As shown in Fig. 3A and Additional file 8: Figure S1A, the intestinal mycobiota of HCC patients were enriched in *Saccharomycetales fam Incertae sedis*, *Candida*, *C. albicans*, *Malasseziomycetes*, *Malasseziales*, *Malasseziaceae*, *Malassezia,* and *Malassezia sp.*; while the gut mycobiome of healthy controls was elevated in *Pleosporaceae*, *Alternaria*, *A. alternata*, *Mucoromycota*, *Mucoromycetes*, *Mucorales*, *Mucoraceae*, *Actinomucor*, *A. elegans*, *Mucor*, and *M. circinelloides*. The distinct taxa at the species level were displayed as relative abundance histograms (Fig. 3B–E). Besides, we also found that there were significant differences in the fungal abundance between the HCC patients and cirrhosis-only patients. The fifteen taxa, including *Saccharomycetes*, *Saccharomycetales*, *Nakaseomyces*, *Nakaseomyces *sp., *Saccharomycetales fam Incertae sedis*, *Candida*, *C. albicans*, *Xylariales*, *Malasseziomycetes*, *Malasseziales*, *Malasseziaceae*, *Malassezia, Malassezia *sp.,* Bipolaris*, and *Bipolaris sp*. were increased in the HCC group (Fig. 3F–N), while *Archaeorhizomycetes*, *Archaeorhizomycetales*, *Archaeorhizomycetaceae*, *Archaeorhizomyces*, *Archaeorhizomyces *sp., *Archaeorhizomycetes *sp., *Hyponectriaceae*, *Monographella*, *M. nivalis*, *Lysurus*, and *L. cruciatus* were highly enriched in the cirrhosis group (Fig. 3F–N). These differentially abundant taxa can be considered potential biomarkers.

### Correlation between gut mycobiota with TNM stage and clinical parameters in HCC patients

**Fig. 4:**
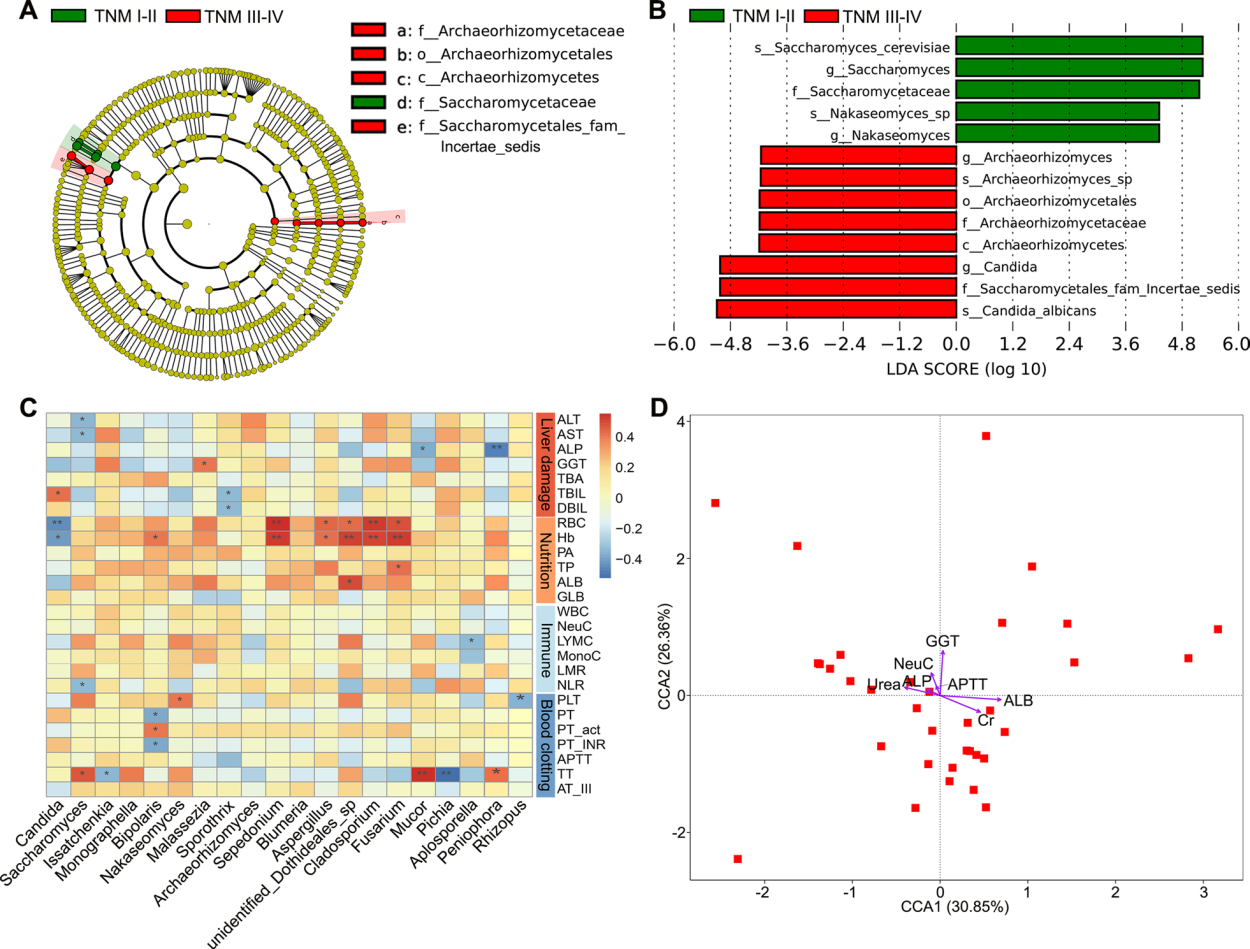
Correlation of gut mycobiota with TNM stage and clinical physiological indicators in HCC patients. **A** Taxonomic cladogram from LEfSe showing differences in fecal taxa of HCC patients with stage III-IV and stage I-II. **B** LDA scores were computed for differentially abundant taxa in the gut fungi of HCC patients with stage III-IV and stage I-II. Length indicates the effect size associated with a taxon. *P* = 0.05 for the Kruskal-Wallis sum-rank test; LDA score > 4; **C** Spearman correlation analysis of fungal taxa and clinical physiological indicators in HCC patients. **D** Canonical correspondence analysis (CCA) of clinical indicators correlated with the fungal profile of patients with HCC. *HCC* hepatocellular carcinoma, *LDA* linear discriminant analysis, *ALT* alanine aminotransferase, *AST* aspartate aminotransferase, *ALP* alkaline phosphatase, *GGT* gamma-glutamyltransferase, *TBIL* total bilirubin, *DBIL* direct bilirubin, *TBA* total bile acid, *RBC* red blood cell, *Hb* hemoglobin, *PA* prealbumin, *TP* total protein, *ALB* albumin, *GLB* globulin, *WBC* white blood cell, *NeuC* neutrophil count, *LYMC* lymphocyte count, *MonoC* monocytes count, *LMR* monocyte-to-lymphocyte ratio, *NLR* neutrophil-lymphocyte ratio, *PLT* platelets, *PT* prothrombin, *PT-act* prothrombin time activity, *PT-INR* the international normalized ratio of prothrombin, *APTT* activated partial thromboplastin time, *TT* thrombin time, *AT-III* antithrombin III, *P < 0.05, **P < 0.01

To further elucidate the relationship between fungal disorders and tumor progression, we used the LEfSe method to estimate differences in fungal taxa between 19 HCC patients with TNM stage III-IV and 15 HCC patients with TNM stage I-II (8th edition of the AJCC/UICC TNM staging system). The clinical characteristics of HCC patients with different TNM stages are detailed in Additional file 6: Table S5. HCC Patients with TNM stage III-IV demonstrated a significantly higher relative abundance of *Saccharomycetales fam Incertae sedis*, *Candida*, *C. albicans*, *Archaeorhizomycetes*, *Archaeorhizomycetales*, *Archaeorhizomycetaceae*, *Archaeorhizomyces*, *Archaeorhizomyces *sp. than the HCC patients with TNM stage I-II. Moreover, *Saccharomycetaceae*, *Nakaseomyces*, *Nakaseomyces *sp., *Saccharomyces*, *S. cerevisiae* were significantly more abundant in HCC patients with TNM stage I-II (Fig. 4A, B).

To understand the relationship between the intestinal fungi and clinical physiological parameters in HCC patients, Spearman analysis was implemented. The top 20 taxa with the highest relative abundance at the genus level were selected for analysis. Data showed that nutritional indicators and liver damage indicators significantly correlated with some fungi (Fig. 4C). For example, the abundance of *Candida* was significantly positively related to the level of TBIL and negatively associated with red blood cell (RBC) and hemoglobin, indicating that *Candida* is associated with impaired liver function and poorer nutritional status. Further, CCA analysis of the clinical parameters screened according to VIF and BioENV showed that albumin and gamma-glutamyltransferase (GGT) were important drivers of fungal distribution in HCC patients (Fig. 4D, Additional file 7: Table S6).

Most of the patients included in this study had a history of HBV infection, so we further analyzed the intestinal fungal differences between 27 patients with HCC and 16 patients with cirrhosis, and those patients had a history of HBV infection. As shown in Additional file 9: Figure S2, the gut mycobiota of HCC patients was enriched in *Candida*, *C*. *albicans*, *Bipolaris*, *Bipolaris *sp., *Nakaseomyces*, *Nakaseomyces *sp., *Sporothrix*,* S*. *ramosissima*, *Malassezia*, *Malassezia *sp., etc*.*, while the gut mycobiota of cirrhosis was elevated in *Monographella*, *M*. *nivalis*, *Lysurus*, *L*. *cruciatus*, *Thermoascus*,* T*. *aurantiacus, *etc*.*

### Classification of the HCC group compared to the non-HCC group

**Fig. 5:**
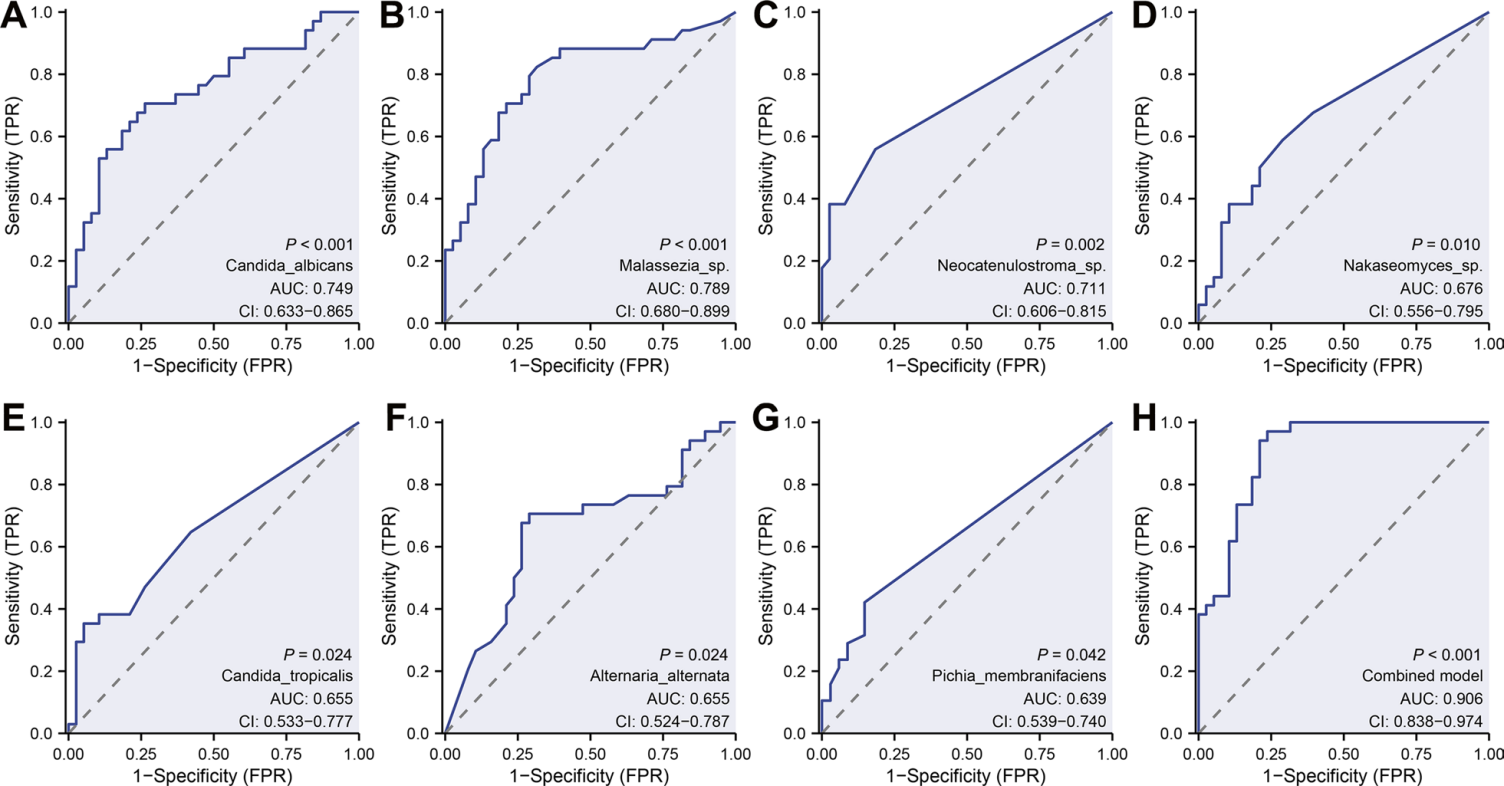
Classification of the HCC group compared to the non-HCC group. ROC curves of *Candida albicans* **A**, *Malassezia sp.* **B**, *Neocatenulostroma sp.* **C**, *Nakaseomyces sp.* **D**, *Candida tropicalis* **E**, *Alternaria alternata* **F**, and *Pichia membranifaciens* **G**. **H** ROC curves analysis to evaluate the classification ability of the gut mycobiome signature (combined *Candida albicans*, *Malassezia sp.*, and *Neocatenulostroma sp.*) in predicting different groups. *ROC* receiver operating characteristic, *AUC* area under the curve

We further explored the diagnostic ability of the top eight fungal microbials that showed the most significant differences among three groups (Fig. 2D, E). The ROC curves displayed diagnostic potential for some of these fungi, including *Candida albicans* (AUC = 0.749, Fig. 5A), *Malassezia sp.* (AUC = 0.789, Fig. 5B), *Neocatenulostroma sp.* (AUC = 0.711, Fig. 5C), *Nakaseomyces sp.* (AUC = 0.676, Fig. 5D), *Candida tropicalis* (AUC = 0.655, Fig. 5E), *Alternaria alternata* (AUC = 0.655, Fig. 5F), and *Pichia membranifaciens* (AUC = 0.639, Fig. 5G). The combined models (*Candida albicans*, *Malassezia sp.* and *Neocatenulostroma sp.*) confirmed the powerful discriminatory ability of intestinal flora in distinguishing HCC patients from healthy controls and cirrhosis (AUC = 0.906, Fig. 5H). However, the above findings are yet to be confirmed by future multicenter studies with large sample sizes.

### Functional classification prediction of the specific taxonomic

**Fig. 6:**
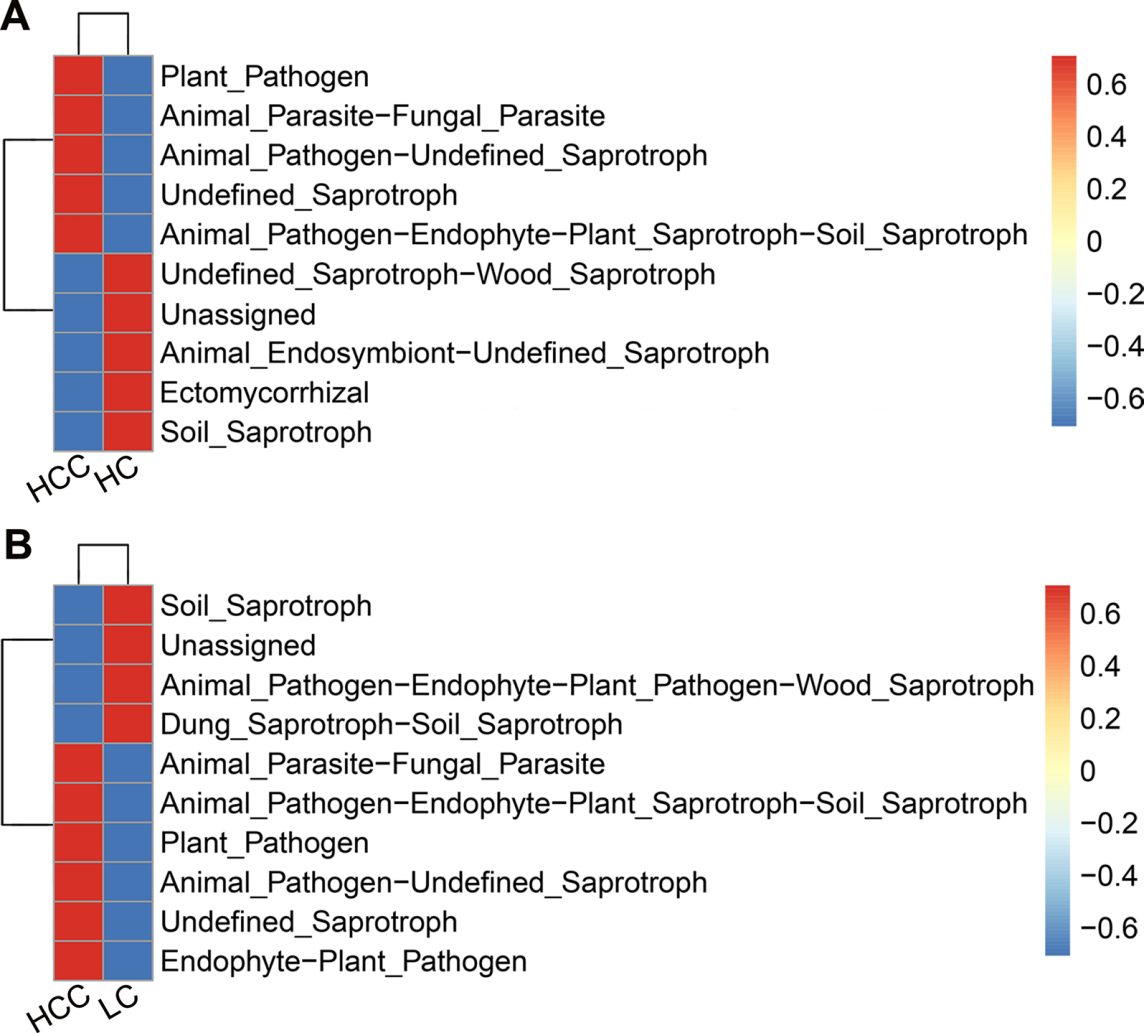
Functional classification predictions. Fungal functional annotations between the HCC and healthy controls **A** and between the HCC and liver cirrhosis **B** were performed by FUNGuild. Fungi were divided into different categories at the Guild levels according to the ways of absorption and utilization of environmental resources. *HCC* hepatocellular carcinoma, *LC* liver cirrhosis, *HC* healthy controls

Because of lacking a powerful tool for annotating the function of fungi, we concentrated on the functional guilds of the fungal microorganisms instead, using FUNGuild. As shown in Fig. 6A, B, the clustered heatmap revealed distinctively differential functions among HCC patients, cirrhotic patients, and healthy controls. Specifically, the pathotrophs, such as plant pathogen, animal pathogen-undefined saprotroph, and animal pathogen-endophyte-plant saprotroph-soil saprotroph, were significantly enriched in the HCC group (Fig. 6A, B); the saprotrophs, including soil saprotroph and dung saprotroph-soil saprotroph, and animal pathogen-endophyte-plant pathogen-wood saprotroph were remarkably increased in the cirrhosis group (Fig. 6B); while the saprotrophs, such as soil saprotroph and undefined saprotroph-wood saprotroph, and ectomycorrhiza were significantly enriched in the healthy controls (Fig. 6A). Thus, our analysis indicates that the symbiotic ecological relationships of gut fungi are altered in patients with HCC and are dominated by pathological parasitism, which can receive nutrients from and adversely affect host cells.

### C. albicans and M. furfur promote the progression of HCC

**Fig. 7:**
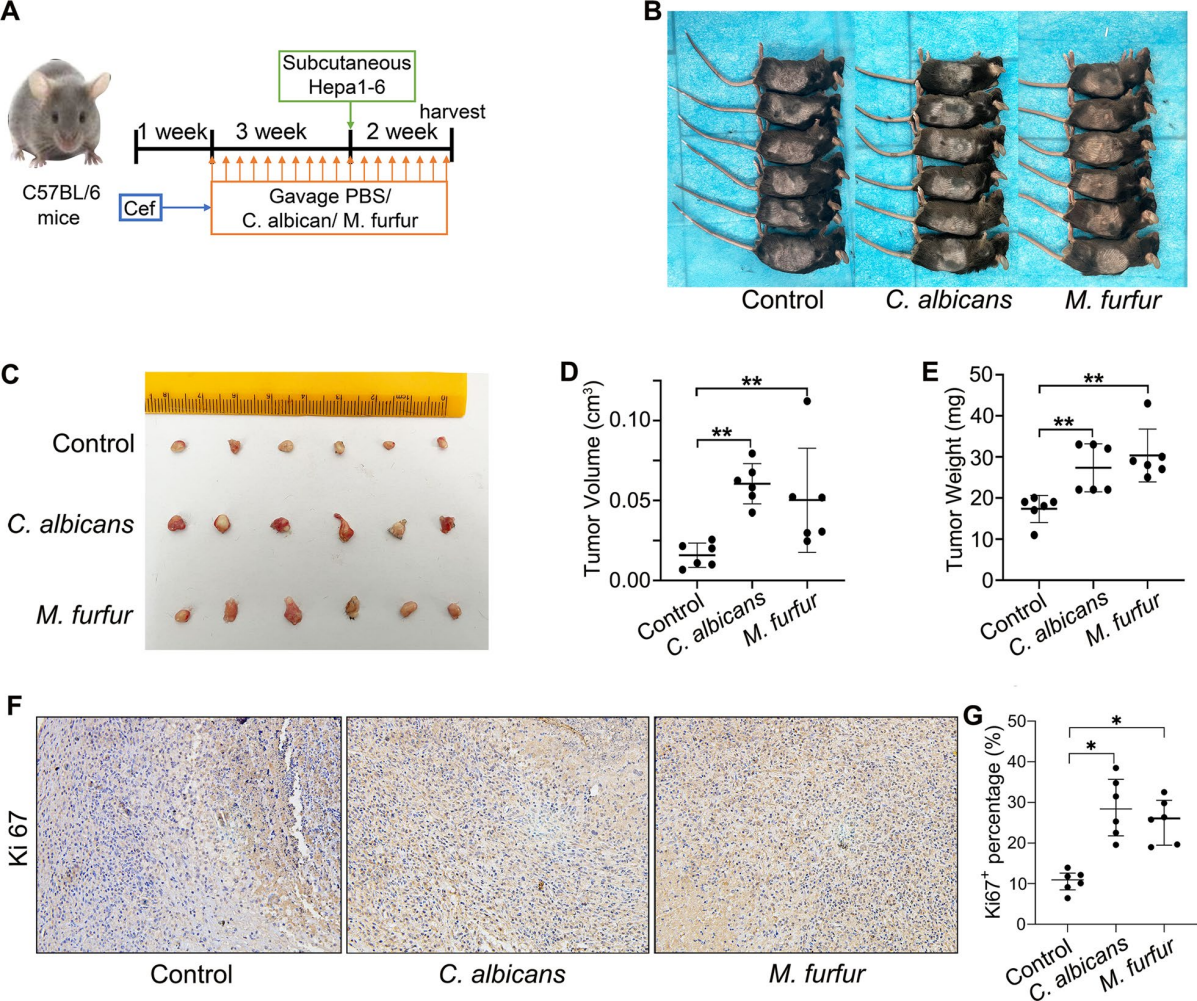
Abnormal colonization of *C. albicans* and *M. furfur* can promote HCC development. **A** Schematic diagram of the oral gavage intervention protocol using phosphate-buffered saline, *C. albicans* or *M. furfur* for C57BL/6 mice (6 mice per group). The images of tumor-bearing mice **B** and tumor masses **C**. Tumor volume **D** and weight **E** were compared among the three groups at the end of the experiment. **F** Representative images of subcutaneous tumor among the three groups immunostained with Ki67 (200 ×). **G** Quantification of Ki67 + cells. *P < 0.05, **P < 0.01, Wilcoxon rank sum test was used

To confirm whether *C. albicans* and *M. furfur* are involved in HCC development, we treated C57BL/6 mice with oral gavage of PBS, *C. albicans*, or *M. furfur* and inoculated them with subcutaneous tumors (Fig. 7A, B). We found that after 2 weeks post exposure, the tumor volume and weight were significantly increased in the *C. albicans* and *M. furfur* groups, while there was no significant difference in the body weight among the three groups (Fig. 7C–E, Additional file 10: Figure S3). IHC staining confirmed that more proliferative cells were observed in the xenografts of the *C. albicans* and *M. furfur* groups as indicated by Ki-67 staining (Fig. 7F, G). These results indicate that abnormal colonization by *C. albicans* and *M. furfur* contributes to HCC development.

## Discussion

Liver cancer is a highly aggressive but silent malignancy because of the tremendous compensating abilities of the liver. In this study, we revealed, for the first time, the presence of intestinal fungal dysbiosis with significant enrichment of opportunistic pathogenic fungi, including *Malassezia*, *Malassezia *sp., *Candida*, and *C. albicans* in HCC patients compared with healthy controls and cirrhotic patients. Among these, the abundance of *C. albicans* was remarkably higher in HCC patients with stage III-IV than in those with stage I-II. The animal experiments confirm that aberrant colonization in the intestine by *C. albicans* and *M. furfur* can promote the development of HCC.

In our study, we found that alpha diversity was significantly lower in cirrhosis compared to healthy controls, which is consistent with the findings of Bajaj et al*.* [32]. They found that gut fungal diversity was significantly lower in 77 outpatients and 66 inpatients with cirrhosis than in 26 control populations. This implies that the sample selection in our study was representative.

Notably, the levels of *Aspergillus flavus* and *Aspergillus parasiticus* (aflatoxin producers) were similar among the three groups. Previous studies have revealed that *Malassezia* usually exists in the skin and has the ability to colonize the gastrointestinal tract [44, 45]. Recently, Aykut et al. [23] were the first to find that *Malassezia* can migrate from the gut to the pancreas and promote pancreatic ductal adenocarcinoma through activation of the mannose-binding lectin and complement-3 pathway. We have found through *Malassezia* colonization experiments that it can promote the progression of liver cancer.

Interestingly, the alterations in *C. albicans* were more prominent as the TNM stage of HCC advanced. Currently, *C. albicans* is by far the most studied opportunistic pathogenic fungi. The carcinogenic effect of *C. albicans* has long been disclosed in multiple cancers [24, 46–50]. For example, Vadovics et al*.* [24] revealed that *C. albicans* accelerated oral cancer development by upregulating the synthesis of matrix metalloproteinases, oncometabolite, and pro-tumor signaling pathways, as well as upregulating prognostic marker genes linked to metastatic events. Zhu and his colleagues [48] reported that the *C. albicans* abundance was remarkably higher in patients with colitis-associated cancer. The increase in *C. albicans* can induce the upregulation of glycolysis in macrophages through the hypoxia-inducible factor (HIF)-1 pathway, triggering an increase in the secretion of interleukin (IL)-7 from macrophages [48]. IL-7 induce IL-22 production in intestinal intrinsic lymphocytes 3, which in turn promotes the proliferation of intestinal epithelial cells and the progression of colitis-associated cancer [48]. By interacting with Dectin-1 in intestinal epithelial cells, *C. albicans* also promote the proliferation of intestinal epithelial cells [47]. In line with these findings, our animal studies also confirm the pro-cancer role of *C. albicans* in the development of HCC.

Besides, the AUC showed that the combined model had very satisfactory diagnostic performance. However, it is worth noting that the sample size included in this study was limited, and it was a single-center study, so it is not possible to conclude from this study that gut fungal flora characteristics can be used as a potential diagnostic tool for HCC, which needs to be explored in a large multi-center sample in the future. It should also be noted that this study only briefly verified the carcinogenic effects of *C. albicans* and *Malassezia*. The detailed carcinogenic mechanisms of intestinal fungi need to be further investigated in order to provide a new theoretical basis for the treatment of HCC.

## Conclusion

This study revealed the presence of intestinal fungal dysbiosis with significant enrichment of opportunistic pathogenic fungi in HCC patients. The animal experiments confirm that aberrant colonization in the intestine by *C. albicans* and *M. furfur* can promote the development of HCC.

## Supplementary Information

**Additional file 1.** Additional detailed experimental methodology.

**Additional file 2: Table S1.** Table of sequence splicing information, data pre-processing statistics, and quality control information for samples. Combined_reads, Tags sequence from stitching; Qualified, raw Tags filtering low quality and short length sequences; Nochime, after filtering the chimeras, the final Tags sequences used for subsequent analysis, i.e. Effective Tags; Uncombined_reads, sequence of reads that cannot be spliced; Percent_combined(%), ratio of reads from splicing to total_reads from the original down; Effective%, number of Effective Tags as a percentage of the number of total_reads.

**Additional file 3: Table S2.** OTUs clustering and species annotation results.

**Additional file 4: Table S3.** The differential taxa between the HCC patients and healthy controls at the genus and species levels. HCC, hepatocellular carcinoma; HC, healthy controls. Wilcoxon rank sum test was used.

**Additional file 5: Table S4.** The differential taxa between the HCC patients and cirrhosis patients at the genus and species levels. HCC, hepatocellular carcinoma; LC, liver cirrhosis. Wilcoxon rank sum test was used.

**Additional file 6: Table S5. **Clinical characteristics of patients with HCC at different TNM stages.

**Additional file 7: Table S6.** CCA analysis of clinical parameters and fungal distribution in patients with HCC.

**Additional file 8: Figure S1. **Taxonomic cladogram. (A) Taxonomic cladogram from LEfSe showing differences in fecal taxa of HCC patients and healthy controls. (B) Taxonomic cladogram from LEfSe showing differences in fecal taxa of HCC patients and cirrhosis patients. HCC, hepatocellular carcinoma; LC, liver cirrhosis; HC, healthy controls.

**Additional file 9: Figure S2.** Differential analysis of fungal communities between the patients with hepatocellular carcinoma (hepatitis B viral infection) and cirrhosis (hepatitis B viral infection). (A) Taxonomic cladogram from LEfSe showing differences in fecal taxa of patients with hepatocellular carcinoma (hepatitis B viral infection) and cirrhosis (hepatitis B viral infection). (B) LDA scores were computed for differentially abundant taxa in the gut fungi of patients with hepatocellular carcinoma (hepatitis B viral infection) and cirrhosis (hepatitis B viral infection). Length indicates the effect size associated with a taxon. *P* = 0.05 for the Kruskal-Wallis sum-rank test; LDA score > 4; LDA, linear discriminant analysis; HBVHCC, patients with hepatocellular carcinoma and hepatitis B virus infection; HBVLC, patients with cirrhosis and hepatitis B virus infection.

**Additional file 10: Figure S3.** The mice weight was compared among the PBS group*, C. albicans *group and *M. furfur *group at the end of the experiment. ^ns^*P* > 0.05, Wilcoxon rank sum test was used.

## Data Availability

The primary accession code for sequence data supporting the findings of this study is PRJNA887395 at the National Center for Biotechnology Information (NCBI). The remaining data can be found within the article, supplemental information, or by contacting the authors directly.
