# Supplementary material for: Characterization of the intestinal fungal microbiome in patients with hepatocellular carcinoma
Source: J Transl Med. 2023 Feb 15;21:126. doi: 10.1186/s12967-023-03940-y (PMC9933289; doi:10.1186/s12967-023-03940-y)
Supplement: Supplementary file 1 — Additional file 1. Additional detailed experimental methodology. [file 12967_2023_3940_MOESM1_ESM.docx]

**Additional file**

**1. PCR amplification**

All PCR reactions were performed with 15 µL of Phusion® High-Fidelity PCR Master Mix (New England Biolabs), 2 µM of forward and reverse primers (ITS2, ITS3-2024F (5’-GCATCGATGAAGAACGCAGC-3’) and ITS4-2409R (5’- TCCTCCGCTTATTGATATGC-3’)), and about 10 ng template DNA. Thermal cycling conditions were as follows: initial denaturation at 98°C for 1 minute; 30 cycles of denaturation at 98°C for 10 seconds, annealing at 50°C for 30 seconds, and elongation at 72°C for 30 seconds; and completed with the final elongation step at 72°C for 5 minutes. Note that steps such as fecal DNA extraction and PCR amplification were carried out in a UV-sterilized biological safety cabin for at least 60 minutes to avoid contamination of environmental DNA. Besides, a negative control was set up for the PCR amplification session. The negative control was to replace the DNA sample in the PCR system with ddH_2_O and keep the rest unchanged in order to see if there was any contamination in the amplification process.

**2. Sequencing data processing**

The data of each sample was split from the sequencing data based on Barcode and PCR amplified primer sequences, and the reads of each sample were merged using FLASH (V1.2.7, http://ccb.jhu.edu/software/FLASH/) [1] after truncating the Barcode and primer sequences, and the splicing sequences were called raw Tags data (Raw Tags). The Raw Tags were then rigorously filtered using fastp software [2] to obtain the high-quality Tags data (Clean Tags). The Tags obtained after the above processing were compared with the species annotation database (https://github.com/torognes/vsearch/) [3] to detect chimera sequences, and then the chimera sequences were removed [4]. The Effective Tags were finally obtained.

**3. OTUs cluster and Species annotation**

Using the Uparse algorithm (Uparse v7.0.1001, http://www.drive5.com/uparse/) [5] to cluster all Effective Tags, the Sequences with ≥97% similarity were assigned to the same OTUs (Operational Taxonomic Units). The sequence with the highest frequency of occurrence in OTUs was selected as the representative sequence of OTUs. For each representative sequence, the Unite Database (https://unite.ut.ee/) [6] was used based on blast algorithm to annotate taxonomic information [7]. To study phylogenetic relationship of different OTUs, and the difference of the dominant species in different samples, multiple sequence alignment was conducted using the MUSCLE software (Version 3.8.31, <http://www.drive5.com/muscle/>) [8]. OTUs abundance information was normalized using a standard sequence number corresponding to the sample with the least sequences. Subsequent analyses of alpha diversity and beta diversity were all performed based on this output normalized data.

**4. Immunohistochemical staining**

The paraffin sections were placed on a stainless shelf and incubated at 70 °C for 80 min in an oven. After xylene deparaffinization twice (20 min each), gradient ethanol hydration (100%, 95%, 85%, 75%, 5%, 5 min each), and deionized water washing. The sections were put on a boil of sodium citrate buffer (pH 9.0) and further boiled for 4 min in a pressure cooker for antigen retrieval. After cooling, the slides were blocked with a 3% hydrogen peroxide solution for 15 min. The sections were incubated overnight at 4°C with an anti-Ki67 antibody (rabbit, abclonal, 1:200) in a humidity box, then incubated with a second antibody. The staining results were visualized using 3,5-diaminobenzidine. Images were obtained with a fluorescence microscope (Olympus BX63, Tokyo, Japan).

**Reference**

1. Magoč T, Salzberg SL. FLASH: fast length adjustment of short reads to improve genome assemblies. Bioinformatics. 2011;27(21):2957-63. Epub 2011/09/10. doi: 10.1093/bioinformatics/btr507. PubMed PMID: 21903629; PubMed Central PMCID: PMCPMC3198573.

2. Bokulich NA, Subramanian S, Faith JJ, Gevers D, Gordon JI, Knight R, et al. Quality-filtering vastly improves diversity estimates from Illumina amplicon sequencing. Nat Methods. 2013;10(1):57-9. Epub 2012/12/04. doi: 10.1038/nmeth.2276. PubMed PMID: 23202435; PubMed Central PMCID: PMCPMC3531572.

3. Rognes T, Flouri T, Nichols B, Quince C, Mahé F. VSEARCH: a versatile open source tool for metagenomics. PeerJ. 2016;4:e2584. Epub 2016/10/27. doi: 10.7717/peerj.2584. PubMed PMID: 27781170; PubMed Central PMCID: PMCPMC5075697.

4. Haas BJ, Gevers D, Earl AM, Feldgarden M, Ward DV, Giannoukos G, et al. Chimeric 16S rRNA sequence formation and detection in Sanger and 454-pyrosequenced PCR amplicons. Genome Res. 2011;21(3):494-504. Epub 2011/01/08. doi: 10.1101/gr.112730.110. PubMed PMID: 21212162; PubMed Central PMCID: PMCPMC3044863.

5. Edgar RC. UPARSE: highly accurate OTU sequences from microbial amplicon reads. Nat Methods. 2013;10(10):996-8. Epub 2013/08/21. doi: 10.1038/nmeth.2604. PubMed PMID: 23955772.

6. Kõljalg U, Nilsson RH, Abarenkov K, Tedersoo L, Taylor AF, Bahram M, et al. Towards a unified paradigm for sequence-based identification of fungi. Mol Ecol. 2013;22(21):5271-7. Epub 2013/10/12. doi: 10.1111/mec.12481. PubMed PMID: 24112409.

7. Altschul SF, Gish W, Miller W, Myers EW, Lipman DJ. Basic local alignment search tool. J Mol Biol. 1990;215(3):403-10. Epub 1990/10/05. doi: 10.1016/s0022-2836(05)80360-2. PubMed PMID: 2231712.

8. Edgar RC. MUSCLE: multiple sequence alignment with high accuracy and high throughput. Nucleic Acids Res. 2004;32(5):1792-7. Epub 2004/03/23. doi: 10.1093/nar/gkh340. PubMed PMID: 15034147; PubMed Central PMCID: PMCPMC390337.
