# Supplementary figures and images for: Characterization of the intestinal fungal microbiome in patients with hepatocellular carcinoma
Source: J Transl Med. 2023 Feb 15;21:126. doi: 10.1186/s12967-023-03940-y (PMC9933289; doi:10.1186/s12967-023-03940-y)

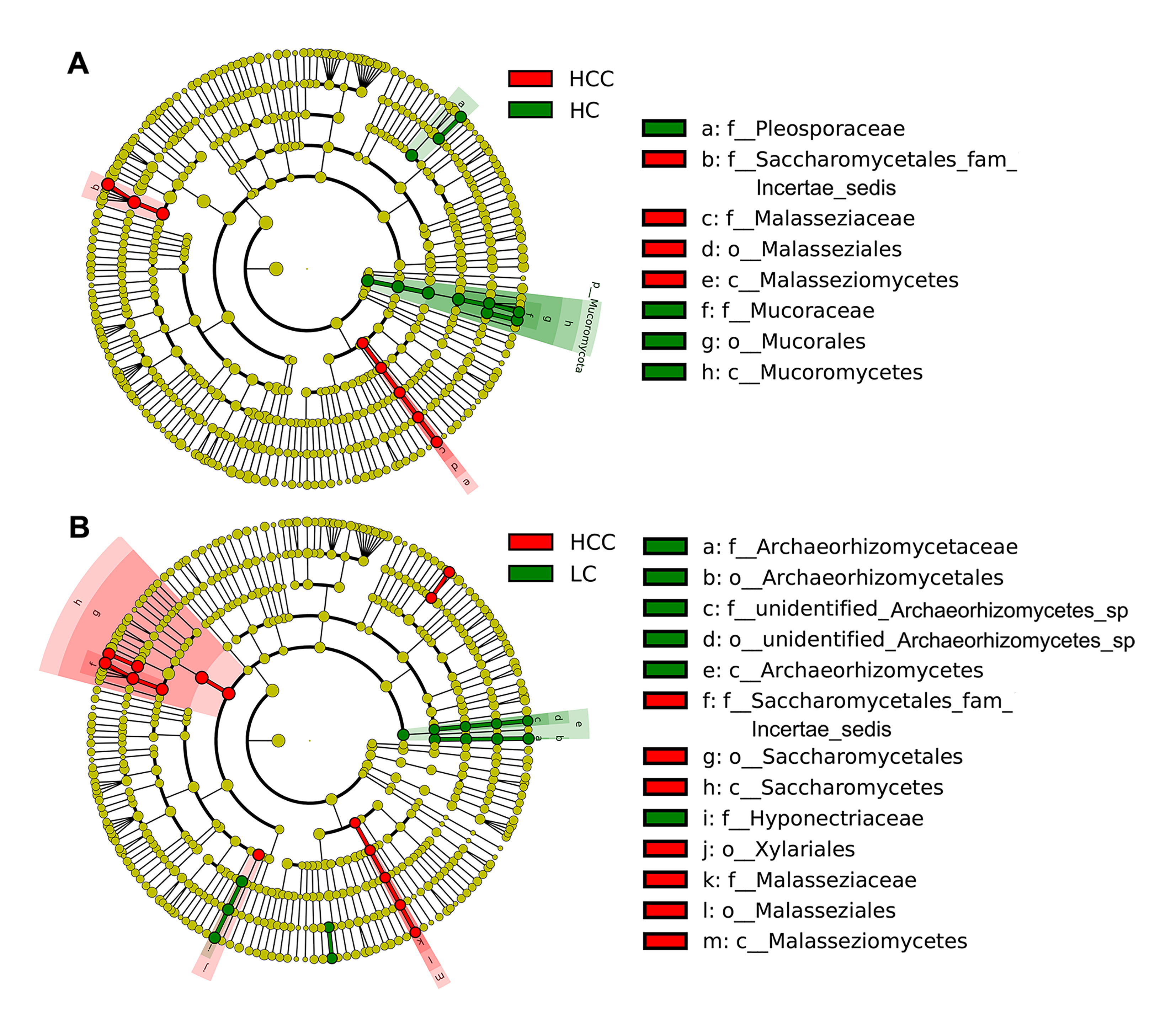

Supplement: Supplementary file 8 — Additional file 8: Figure S1. Taxonomic cladogram. (A) Taxonomic cladogram from LEfSe showing differences in fecal taxa of HCC patients and healthy controls. (B) Taxonomic cladogram from LEfSe showing differences in fecal taxa of HCC patients and cirrhosis patients. HCC, hepatocellular carcinoma; LC, liver cirrhosis; HC, healthy controls. [file 12967_2023_3940_MOESM8_ESM.tif]

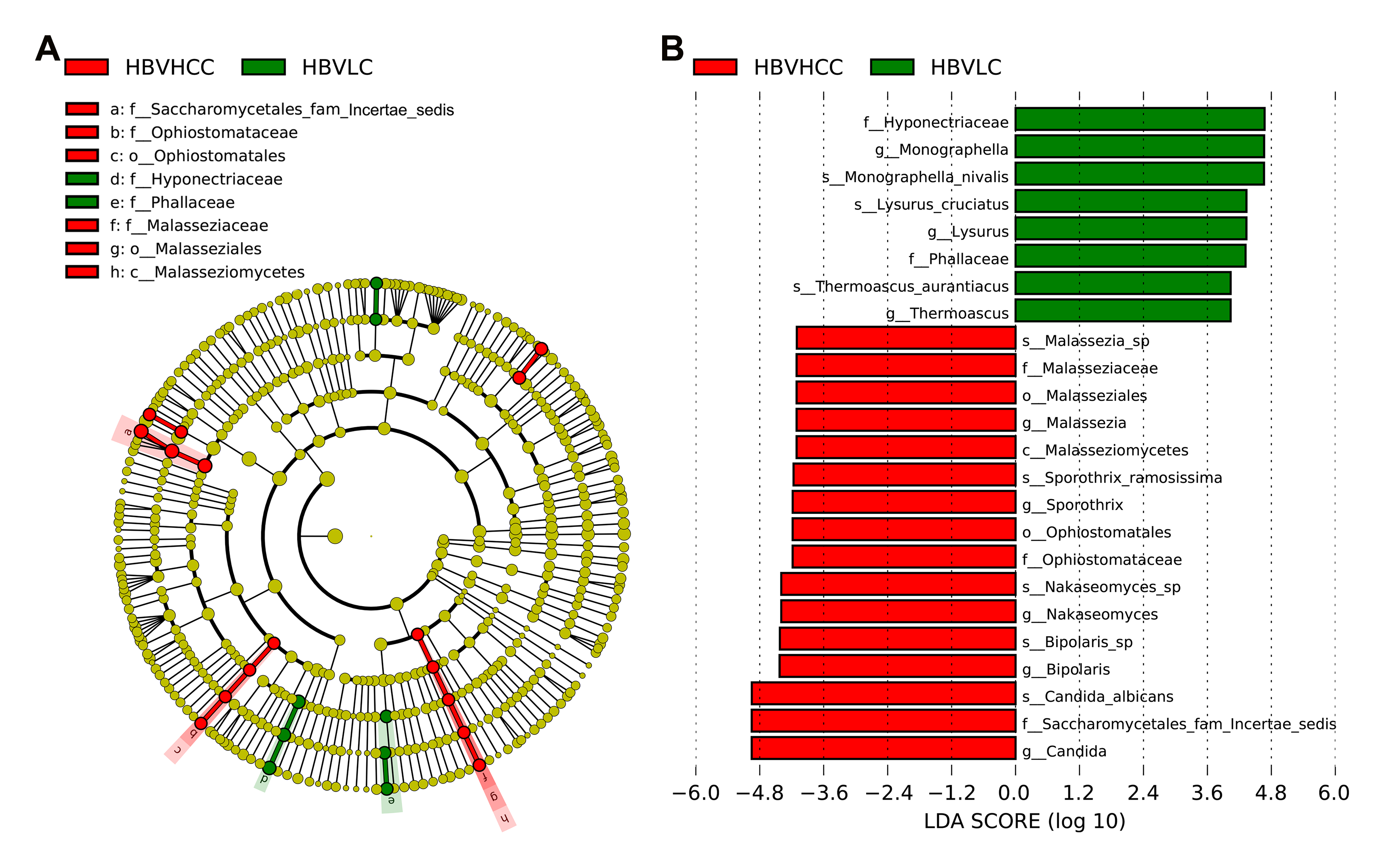

Supplement: Supplementary file 9 — Additional file 9: Figure S2. Differential analysis of fungal communities between the patients with hepatocellular carcinoma (hepatitis B viral infection) and cirrhosis (hepatitis B viral infection). (A) Taxonomic cladogram from LEfSe showing differences in fecal taxa of patients with hepatocellular carcinoma (hepatitis B viral infection) and cirrhosis (hepatitis B viral infection). (B) LDA scores were computed for differentially abundant taxa in the gut fungi of patients with hepatocellular carcinoma (hepatitis B viral infection) and cirrhosis (hepatitis B viral infection). Length indicates the effect size associated with a taxon. P = 0.05 for the Kruskal-Wallis sum-rank test; LDA score > 4; LDA, linear discriminant analysis; HBVHCC, patients with hepatocellular carcinoma and hepatitis B virus infection; HBVLC, patients with cirrhosis and hepatitis B virus infection. [file 12967_2023_3940_MOESM9_ESM.tif]

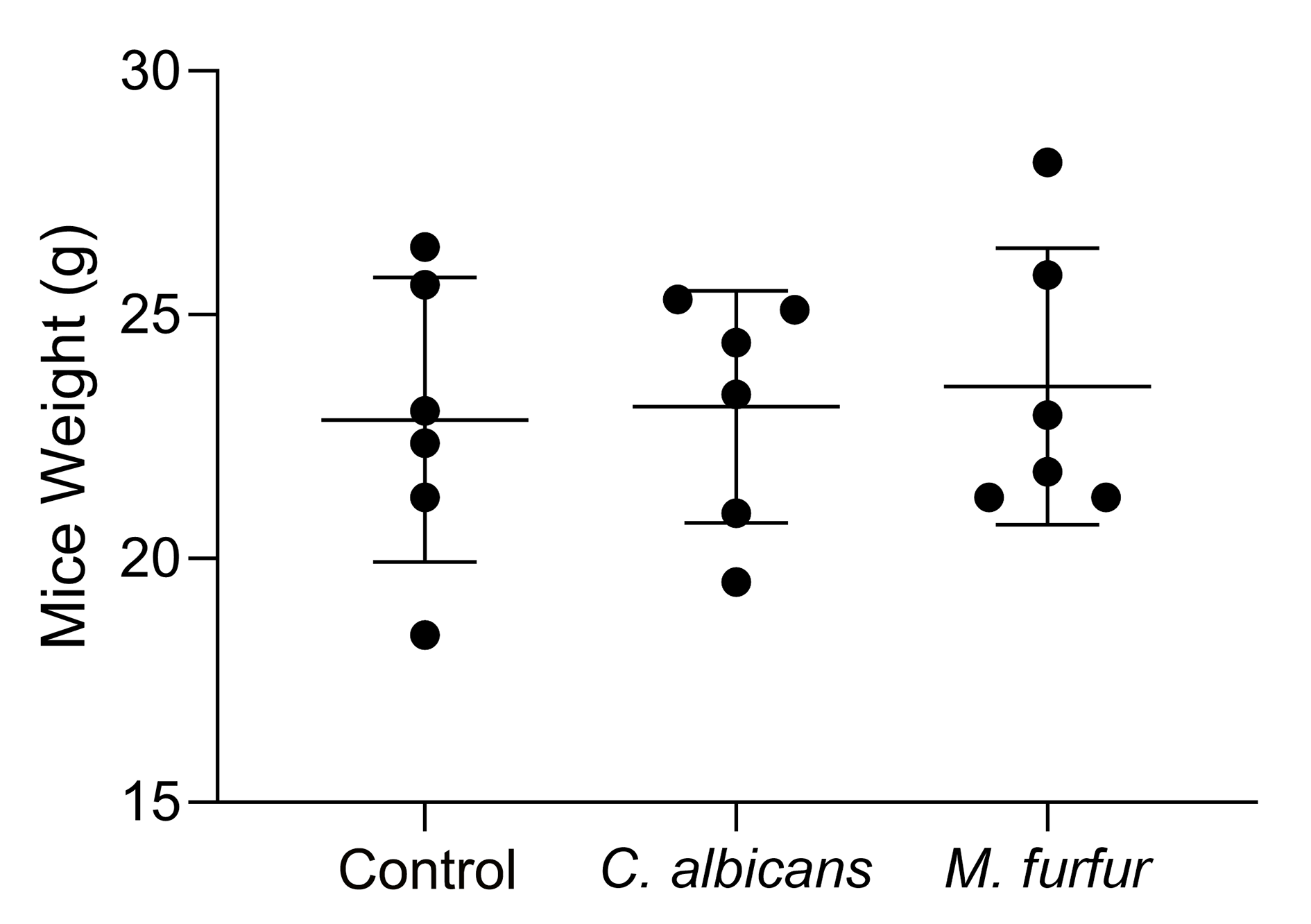

Supplement: Supplementary file 10 — Additional file 10: Figure S3. The mice weight was compared among the PBS group, C. albicans group and M. furfur group at the end of the experiment. nsP > 0.05, Wilcoxon rank sum test was used. [file 12967_2023_3940_MOESM10_ESM.tif]
